# Thirty-day clinical outcomes, early recovery, and healthcare resource use after planned ambulatory vs. conventional inpatient laparoscopic cholecystectomy: a propensity-score overlap-weighted retrospective cohort study

**DOI:** 10.3389/fsurg.2026.1962424

**Published:** 2026-10-16

**Authors:** Yi Hao, Yi Wang, Qinyang Du, Rongshen Guan, Qirui Zhang, Gaopeng Li

**Affiliations:** Department of Hepatobiliary Surgery, Third Hospital of Shanxi Medical University, Shanxi Bethune Hospital, Shanxi Academy of Medical Sciences, Tongji Shanxi Hospital, Taiyuan, China

**Keywords:** ambulatory surgery, gallstones, healthcare resource use, laparoscopic cholecystectomy, overlap weighting, propensity score

## Abstract

**Background:**

Real-world comparisons of planned ambulatory and conventional inpatient laparoscopic cholecystectomy are vulnerable to patient selection and organisational differences. We used propensity score overlap weighting to compare 30-day safety, early recovery, and recorded healthcare resource use between the two planned pathways.

**Methods:**

This single-centre retrospective cohort included 223 adults who underwent elective laparoscopic cholecystectomy for benign gallstone-related disease at Shanxi Bethune Hospital between May 2025 and June 2026. Patients were classified by their preoperative plan as ambulatory (defined as arrival on the day of surgery and intended discharge within 24 h) or conventional inpatient care. The primary outcome was a 30-day adverse clinical composite comprising Clavien–Dindo grade ≥2 complications, unplanned emergency department visits, readmission, endoscopic retrograde cholangiopancreatography or another intervention, reoperation, or death. Overlap weights balanced preoperative characteristics, and 1:1 propensity score matching served as a sensitivity analysis.

**Results:**

The ambulatory and inpatient groups included 78 and 145 patients, respectively. All included covariates demonstrated absolute standardised mean differences below 0.10 after overlap weighting. Weighted risks of the 30-day composite were 3.8% and 10.2%, giving a risk ratio of 0.37 (95% confidence interval, 0.02 to 1.25) and a risk difference of −6.4 percentage points (−13.8 to 1.4; *P* = 0.100). The ambulatory pathway was associated with 70.3 fewer postoperative hours and 100.0 fewer total hospital hours. Ambulation and oral intake occurred 5.7 and 6.9 h earlier, discharge pain was 1.13 points lower, and recorded index-admission hospital charges were CNY 5,200 lower; all *P* < 0.001. Seven ambulatory patients (9.0%) had pathway failure. Results from 59 matched pairs were directionally consistent.

**Conclusions:**

Among patients with a realistic possibility of receiving either pathway, planned ambulatory care was not associated with an increase in the 30-day adverse clinical composite and was associated with shorter hospital stay and lower recorded healthcare resource utilisation. However, important determinants of pathway selection were incompletely measured. The single-centre observational design, residual confounding, and small number of primary events preclude causal interpretation or claims of safety equivalence.

## Introduction

Gallstone disease is a common digestive disorder that can cause biliary colic, acute cholecystitis, biliary pancreatitis and recurrent hospital attendances. Laparoscopic cholecystectomy remains the principal surgical treatment for symptomatic gallstones and chronic calculous cholecystitis. Current guidelines emphasise safe anatomical dissection and prevention of bile duct injury at the centre of operative care (1–8). Advances in minimally invasive surgery, short-acting anaesthetic agents, multimodal analgesia and enhanced recovery pathways have allowed elective procedures to transition from multi-day inpatient admissions to ambulatory pathways.

More than two decades of evidence have accumulated supporting ambulatory or outpatient laparoscopic cholecystectomy. Randomised trials, cohort studies and meta-analyses generally suggest that selected patients can be safely discharged on the day of surgery or after a short observation period without a clear increase in readmission or serious complications (9–19). Definitions of ambulatory care, eligibility, discharge readiness, and follow-up nevertheless vary across studies. Reported outcomes therefore reflect local bed capacity, anaesthetic processes and post-discharge support as well as the operation itself.

Successful ambulatory discharge usually depends on several linked factors. Age and comorbidity influence baseline risk, while the operative start time determines the available observation window. Operative complexity, pain, and postoperative nausea and vomiting directly affect recovery. Social support, travel distance, and access to unplanned overnight care are also critical determinants (20–23). These factors shape both pathway assignment and postoperative outcomes, creating substantial confounding in real-world comparisons.

Propensity score methods can address measured confounding, but conventional inverse probability weighting may produce extreme weights when scores approach zero or one. Overlap weighting instead emphasises patients whose preoperative characteristics make either pathway plausible and uses bounded weights to limit extreme-score influence (24–27). This target population resembles patients for whom clinicians genuinely have a pathway choice, which is the question of practical interest.

Chinese single-centre programmes still require evidence that integrates safety, recovery and healthcare resource utilisation. We therefore compared planned ambulatory and conventional inpatient laparoscopic cholecystectomy using routine care and 30-day follow-up data. We hypothesised that, after balancing measured preoperative characteristics, ambulatory care would not increase the 30-day adverse composite and would be associated with earlier recovery milestones, a shorter hospital stay, and lower recorded index-admission hospital charges.

## Materials and methods

### Study design

We conducted a single-centre retrospective cohort study using routine clinical records and 30-day follow-up data from the Department of Hepatobiliary and Pancreatic Surgery at Shanxi Bethune Hospital. The institutional ambulatory laparoscopic cholecystectomy programme had been introduced in January 2025, before the start of the analytic study period. Eligible procedures were performed between May 2025 and 8 June 2026. The final 30-day follow-up assessment for the analytic cohort was completed on 9 July 2026, and the analytic database was locked on 10 July 2026. Thus, all patients included in the 30-day safety analysis had completed the required follow-up before database lock. A total of 317 consecutive potentially eligible cholecystectomy procedures were screened. After application of the pre-specified eligibility criteria, 94 patients were excluded, and 223 patients formed the final analytic cohort. The patient was the unit of analysis, and each patient was included in the cohort once.

Reporting was guided by the STROBE statement (28). The hospital ethics committee approved the study (YXLL-2025–055) and waived written informed consent because existing clinical data were used.

### Participants

Eligible patients were aged at least 18 years and had a diagnosis of cholelithiasis, chronic calculous cholecystitis, recurrent biliary colic, or recovering mild acute cholecystitis. All participants underwent elective laparoscopic cholecystectomy and had complete perioperative and 30-day outcome data. Consecutive eligible patients planned for either ambulatory or conventional inpatient care were included according to an intention-to-treat principle to avoid immortal time or per-protocol selection bias.

We excluded 11 patients who underwent planned open surgery, 23 with suspected or confirmed gallbladder malignancy, and 17 with known common bile duct stones requiring concurrent common bile duct exploration or perioperative endoscopic retrograde cholangiopancreatography (ERCP). A further 26 patients were excluded because of acute cholangitis, severe acute cholecystitis, or acute pancreatitis, 2 because they underwent another concurrent procedure; and 15 because complete 30-day outcome ascertainment was not available. These mutually exclusive exclusions accounted for 94 of the 317 screened procedures, leaving 223 patients in the final analytic cohort. Intraoperative conversion to open cholecystectomy after laparoscopic access was not an exclusion criterion and remained an important safety outcome analysed according to the original planned pathway.

### Pathway assignment and perioperative care

Patients were classified by their preoperative management plan rather than their final length of stay. The ambulatory pathway involved no conventional preoperative admission, arrival on the day of surgery and intended discharge within 24 postoperative hours. Patients requiring longer observation for pain, nausea, high or bilious drainage or delayed recovery remained in the primary ambulatory group and were recorded as pathway failures. The conventional inpatient group followed the hospital's standard admission, operative, and discharge pathways.

Surgeons and anaesthesiologists jointly assessed ambulatory candidates. Screening considered age, ASA physical status classification, control of chronic medical conditions, functional capacity, the stage of gallbladder disease and anticipated operative complexity. Patients and families also needed to understand the pathway and have reliable adult caregiver support, telephone contact, and established emergency return arrangements. Patients with ASA class III were considered only when comorbidities were stable after multidisciplinary preoperative evaluation. These eligibility assessments were made as part of routine clinical care. Functional capacity, anticipated operative complexity, social support, pathway understanding, telephone availability, and emergency return feasibility were not recorded using standardised, validated instruments and therefore could not all be incorporated into the retrospective propensity score model.

Surgical, anaesthetic and nursing teams jointly assessed discharge readiness. Patients needed clear consciousness, stable vital signs, pain controlled with oral or routine analgesia, and absent or controlled nausea and vomiting. They were also required to tolerate oral fluids or a light diet, mobilise, and void. Active wound bleeding, progressive abdominal pain, fever, jaundice, or other red-flag clinical signs prevented discharge. Patients who did not meet these criteria remained under observation or were admitted to a conventional ward. Both groups underwent laparoscopic cholecystectomy under general anaesthesia with routine postoperative monitoring.

### Data collection and quality control

Preoperative variables included age, sex, body mass index, smoking history, ASA physical status class, hypertension, diabetes mellitus, cardiopulmonary disease, liver or renal dysfunction, antithrombotic therapy and previous abdominal surgery. We also recorded the primary diagnosis, previous biliary episodes, biliary-related emergency department visits or admissions, gallbladder wall thickness, impacted gallbladder neck calculi, white blood cell count, C-reactive protein, alanine aminotransferase, total bilirubin, creatinine and surgeon experience. These variables were available before pathway assignment and informed the baseline covariate balance or the propensity model.

Operative and recovery measures included operative start time, prophylactic antibiotic administration, anaesthesia duration, operating time, blood loss, operative difficulty and intraoperative adverse events. Drain placement, conversion to open laparotomy, post-anaesthesia care unit length of stay, discharge pain scores, rescue analgesia requirements, postoperative nausea and vomiting, time to ambulation, time to oral intake, time to discharge readiness, postoperative length of stay, and total hospital length of stay were also collected. Recorded billing variables comprised medication charges, surgery and anaesthesia charges, and aggregate recorded index-admission hospital charges in Chinese yuan (CNY). Operative difficulty was graded using the Sugrue operative difficulty grading score (0–10), with scores of <2 indicating mild, 2–4 moderate, 5–7 severe, and 8–10 extreme difficulty (29). Pain intensity was assessed using the 0–10 numeric rating scale (NRS), where 0 represents “no pain” and 10 represents “the worst imaginable pain” (30). Pain was evaluated at predetermined time points: at PACU admission, 4 h postoperatively, at 08:00 each postoperative morning, and immediately before completion of the discharge readiness checklist.

Thirty-day outcomes were ascertained from institutional electronic records, outpatient and emergency department encounters, readmissions, and postoperative follow-up, including telephone or nursing review. Complication diagnoses and Clavien–Dindo grades were extracted as recorded in the source dataset (31, 32). The dataset lacked sufficient information to quantify outcome ascertainment separately through institutional records, outpatient visits, and telephone follow-up, or to describe the timing and standardisation of telephone contacts. Systematic enquiry about care received at other hospitals and blinded outcome assessment could not be verified from the available data. Recorded primary outcome status and propensity score model covariates were complete within the final analytic cohort; thus, no missing-data imputation was performed. This completeness refers strictly to the recorded analytic variables and does not establish comprehensive capture of all postoperative adverse events.

### Outcomes

The primary outcome was a 30-day adverse clinical composite endpoint specified for this retrospective analysis before the principal pathway-specific outcome comparisons were performed. It was not a prospectively registered trial outcome. The composite outcome was deemed to have occurred if a patient experienced any of the following within 30 days of surgery: Clavien–Dindo grade ≥2 complication, an unplanned emergency department visit, an unplanned readmission, ERCP or another endoscopic or percutaneous intervention, reoperation, or death within 30 days. Each patient was counted once in the composite, irrespective of the number of component events. A missing complication diagnosis or grade did not preclude inclusion in the composite when another component criterion was met.

Secondary safety outcomes included any complication, a Clavien–Dindo grade ≥2 complication, emergency department attendance, readmission, postoperative endoscopic or percutaneous intervention, reoperation, intraoperative conversion to open cholecystectomy, bile duct injury and 30-day mortality. Recovery outcomes covered discharge pain scores, rescue analgesia requirements, postoperative nausea and vomiting, post-anaesthesia care unit length of stay, and functional recovery milestones. Resource utilisation outcomes included postoperative and total hospital lengths of stay, recorded index-admission hospital charges, and unplanned healthcare encounters within 30 days. Ambulatory pathway failure and its underlying aetiologies were described only within the ambulatory group.

Pain assessment was performed at identical scheduled time points for both groups (PACU admission, 4 h postoperatively, 08:00 on each postoperative morning, and immediately before discharge), and the discharge readiness checklist (tolerance of oral intake, unaided ambulation, pain controlled with oral analgesia, and absence of surgical complications) was applied identically to both groups. However, because the ambulatory group was discharged on the day of surgery and the conventional group on postoperative day 1 or later, discharge pain scores (0–10 numeric rating scale, recorded immediately before discharge) were obtained at different postoperative time points and are therefore not directly comparable between groups. Monetary values were analysed from the hospital billing perspective and represent recorded index-admission hospital charges rather than hospital cost-accounting expenditures, reimbursement amounts, or a comprehensive economic evaluation. The available billing categories comprised medication charges and surgical and anaesthetic charges; the aggregate recorded hospital charge was the sum of these available categories. More granular item-level components within these categories were not available. The recorded amounts did not comprehensively include ward or nursing charges, diagnostic imaging, laboratory testing, administrative overhead, post-discharge follow-up, or patient-borne costs. The between-group difference also reflected the institutional tariff structure: the ambulatory pathway was billed under the day surgery tariff, whereas the conventional pathway was billed under the inpatient tariff, which included additional charges related to extended post-anaesthesia care and postoperative monitoring.

### Propensity scores and statistical analysis

The patient was the independent unit of analysis. Continuous baseline variables were summarised as mean ± standard deviation for balance assessment, and categorical variables as counts and percentages. Unadjusted outcomes were described using medians with interquartile ranges or counts with percentages. Baseline characteristics were assessed with standardised mean differences rather than hypothesis tests because balance diagnostics concern the magnitude of imbalance rather than sample-size-dependent *P*-values.

A multivariable logistic regression model estimated the probability of planned ambulatory care using the recorded preoperative covariates listed above. The model therefore adjusted for measured baseline differences but could not account for pathway-selection determinants that were not systematically recorded, including structured functional status, anticipated technical complexity, social support and pathway-readiness measures. Operating time, estimated blood loss, drain placement, operative difficulty, pain, and postoperative nausea and vomiting were not included in the propensity score model because they occurred or became evident after pathway assignment and were therefore not appropriate baseline confounders (i.e. post-baseline mediating or collateral variables). Calendar time and individual surgeon identity were not included in the propensity model because exact operation dates and surgeon identifiers were not available at the patient level. Surgeon experience in years was retained as a measured preoperative covariate but was not considered a substitute for surgeon-specific practice patterns. Calendar-period pathway counts were therefore summarised descriptively. Overlap weights were 1-e(X) for ambulatory patients and e(X) for inpatients, where e(X) was the ambulatory propensity score. The target estimand was the average treatment effect in the overlap population.

Absolute standardised mean differences assessed covariate balance, with values below 0.10 considered acceptable. We also examined propensity score and weight ranges and calculated effective sample sizes. Binary outcomes were reported as weighted risks, risk ratios and risk differences. Continuous outcomes were reported as weighted means and adjusted mean differences. Confidence intervals were obtained from 5,000 patient-level non-parametric bootstrap samples, with the propensity model refitted and the outcome analysis repeated in each resample. The reported 95% confidence intervals were percentile intervals defined by the empirical 2.5th and 97.5th percentiles of the bootstrap distribution. Two-sided *P*-values were derived from the bootstrap standard errors. The timing of primary outcome definition is described in the Outcomes section. Secondary analyses were exploratory and were not adjusted for type I error multiplicity. No non-inferiority margin was specified, and the study was not designed to establish safety equivalence or non-inferiority. Continuous covariates were entered into the propensity score model as untransformed linear terms. Multi-category variables were represented by indicator variables, and no spline, polynomial, or interaction terms were included in the primary propensity score model. Overlap weights were 1−e(X) for ambulatory patients and e(X) for inpatients. The resulting estimand was the average treatment effect in the overlap population and therefore applies to patients with a realistic probability of receiving either pathway rather than to all patients undergoing laparoscopic cholecystectomy. Complete diagnostics for the propensity score model, including covariate coding and functional forms (Panel A), model stability and goodness-of-fit metrics (Panel B), pre- and post-weighting covariate balance for both overlap weighting and 1:1 matching (Panel C), bootstrap performance details (Panel D), and matching specifications (Panel E), are provided in Supplementary Table S3.

A sensitivity analysis used 1:1 nearest-neighbour matching without replacement and a calliper of 0.20 standard deviations of the propensity score logit. It repeated the primary composite, postoperative length of stay, total hospital length of stay, and recorded index-admission hospital charge analyses. We included all available eligible cases and did not perform an *a priori* sample size calculation. The primary statistical analysis used Python 3.12.13, while data visualisation and final figures used R 4.5.2. The study flow diagram is shown in Figure 1.

**Figure 1:**
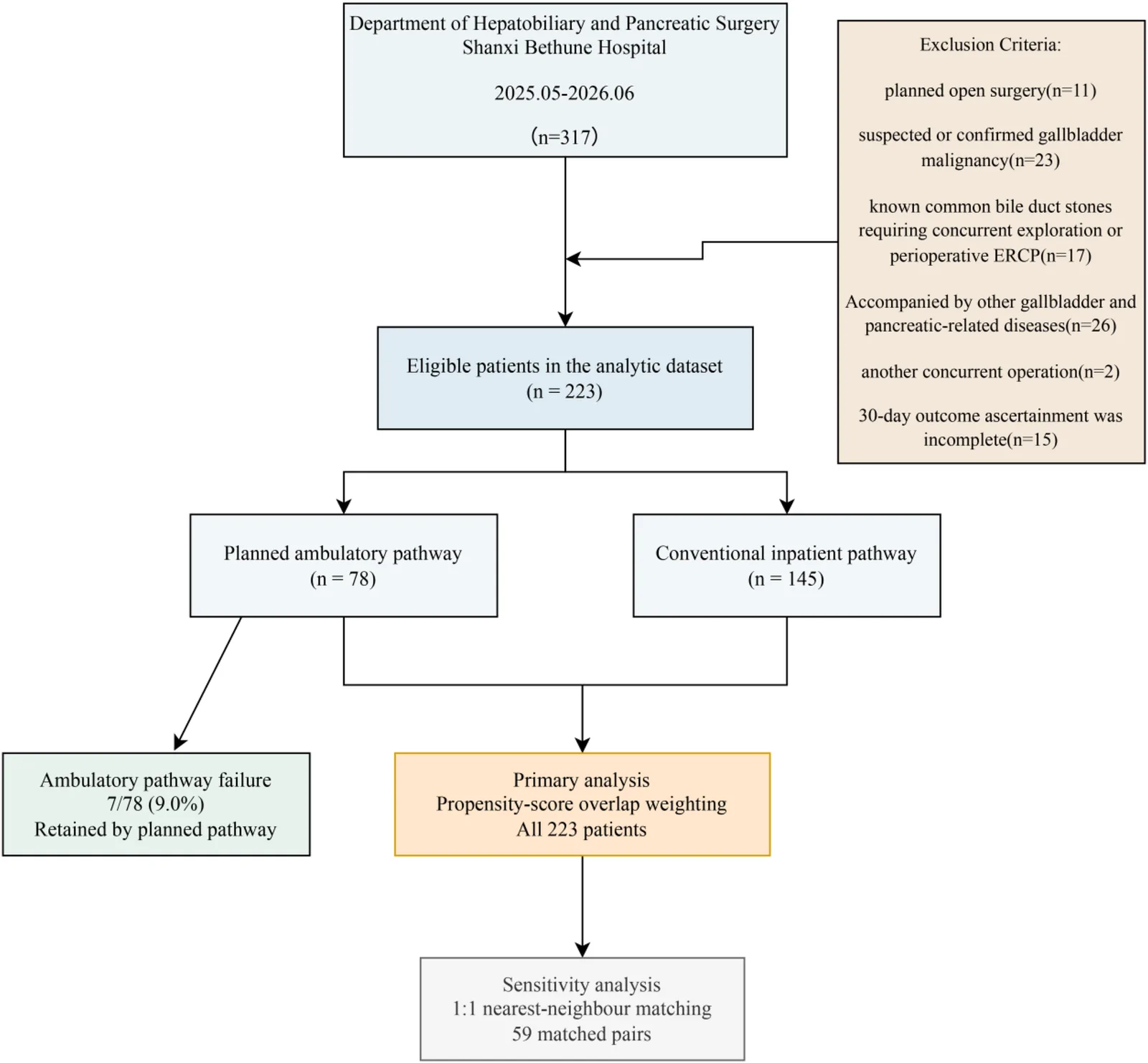
Cohort allocation and analytic workflow. The analytic dataset included 223 eligible patients classified by their preoperative clinical plan as ambulatory care (*n* = 78) or conventional inpatient care (*n* = 145). Seven ambulatory pathway failures remained in their planned group. Overlap weighting used all patients, and sensitivity analysis produced 59 matched pairs.

## Results

### Study population and covariate balance

The analytic cohort comprised 78 patients assigned to planned ambulatory care and 145 assigned to conventional inpatient care. Clear selection patterns were present before weighting. Uncomplicated cholelithiasis was more common in the ambulatory group, whereas chronic calculous cholecystitis and resolving acute cholecystitis were less common. Several comorbidities and previous biliary-related interventions were also less frequent among ambulatory patients. The largest absolute standardised mean difference in Table 1 was 0.566, making unadjusted outcome attribution inappropriate. The ambulatory laparoscopic cholecystectomy programme had been introduced in January 2025. Pathway utilisation varied over the available calendar periods (Supplementary Table S1). From May to July 2025, 13 of 49 procedures (26.5%) were planned ambulatory procedures; this proportion was 26 of 57 (45.6%) from August to October 2025, 12 of 31 (38.7%) from November 2025 to January 2026, and 27 of 86 (31.4%) from February to June 2026. Thus, ambulatory pathway uptake varied over time but did not show a simple monotonic increase across the study period.

**Table 1:** Baseline characteristics before and after overlap weighting.

Variable	Ambulatory, unweighted	Inpatient, unweighted	Unweighted SMD	Ambulatory, weighted	Inpatient, weighted	Weighted SMD
Age, years	51.8 ± 9.6	54.0 ± 12.2	−0.202	52.9 ± 9.7	52.9 ± 11.7	0.000
BMI, kg/m^2^	24.5 ± 2.7	24.9 ± 2.6	−0.150	24.6 ± 2.8	24.6 ± 2.3	0.000
Previous biliary episodes	1.1 ± 1.1	1.3 ± 1.3	−0.159	1.2 ± 1.1	1.2 ± 1.2	0.000
Gallbladder wall thickness, mm	3.3 ± 0.8	3.4 ± 0.8	−0.103	3.3 ± 0.8	3.3 ± 0.8	0.000
White-cell count, ×10⁹/L	7.2 ± 1.4	7.4 ± 1.9	−0.151	7.2 ± 1.5	7.2 ± 1.8	0.000
C-reactive protein, mg/L	8.1 ± 3.5	9.1 ± 8.0	−0.170	8.2 ± 3.6	8.2 ± 6.6	0.000
Alanine aminotransferase, U/L	29.3 ± 11.2	31.5 ± 14.0	−0.167	30.4 ± 11.6	30.4 ± 13.6	0.000
Total bilirubin, μmol/L	13.6 ± 3.0	14.3 ± 5.5	−0.171	13.7 ± 3.0	13.7 ± 5.6	0.000
Creatinine, μmol/L	67.5 ± 12.9	68.8 ± 14.3	−0.090	68.2 ± 13.0	68.2 ± 13.9	0.000
Surgeon experience, years	10.3 ± 3.5	9.8 ± 3.2	0.139	9.8 ± 3.4	9.8 ± 3.3	0.000
Male sex	27 (34.6%)	59 (40.7%)	−0.126	36.4%	36.4%	0.000
Smoking history	8 (10.3%)	23 (15.9%)	−0.167	10.7%	10.7%	0.000
Previous abdominal surgery	12 (15.4%)	34 (23.4%)	−0.205	19.7%	19.7%	0.000
Hypertension	19 (24.4%)	46 (31.7%)	−0.165	26.3%	26.3%	0.000
Diabetes	7 (9.0%)	24 (16.6%)	−0.229	12.1%	12.1%	0.000
Cardiopulmonary disease	4 (5.1%)	18 (12.4%)	−0.260	7.0%	7.0%	0.000
Liver/renal dysfunction	2 (2.6%)	10 (6.9%)	−0.205	4.0%	4.0%	0.000
Anticoagulant or antiplatelet use	3 (3.8%)	12 (8.3%)	−0.186	5.7%	5.7%	0.000
Previous biliary emergency visit	19 (24.4%)	47 (32.4%)	−0.179	28.4%	28.4%	0.000
Previous biliary admission	10 (12.8%)	31 (21.4%)	−0.229	16.4%	16.4%	0.000
Impacted gallbladder neck stone	6 (7.7%)	25 (17.2%)	−0.292	11.7%	11.7%	0.000
ASA class I	15 (19.2%)	21 (14.5%)	0.127	16.2%	16.2%	0.000
ASA class II	51 (65.4%)	94 (64.8%)	0.012	65.3%	65.3%	0.000
ASA class III	12 (15.4%)	30 (20.7%)	−0.138	18.5%	18.5%	0.000
Diagnosis: cholelithiasis	43 (55.1%)	41 (28.3%)	0.566	43.7%	43.7%	0.000
Diagnosis: chronic calculous cholecystitis	12 (15.4%)	57 (39.3%)	−0.557	21.8%	21.8%	0.000
Diagnosis: recurrent biliary colic	21 (26.9%)	33 (22.8%)	0.096	30.0%	30.0%	0.000
Diagnosis: recovering mild acute cholecystitis	2 (2.6%)	14 (9.7%)	−0.299	4.5%	4.5%	0.000

Continuous variables are mean ± standard deviation. Categorical variables are counts (percentages) before weighting and weighted percentages after weighting. SMD, standardised mean difference. An absolute SMD below 0.10 indicates acceptable covariate balance.

Propensity scores ranged from 0.076 to 0.917 in the ambulatory group and from 0.002 to 0.793 in the inpatient group. The region of overlap provided sufficient common support for comparison. Overlap weights ranged from 0.002 to 0.924. After weighting, every included covariate had an absolute standardised mean difference below 0.10. Effective sample sizes were 65.1 and 92.7 for the ambulatory and inpatient groups, respectively. Figure 2 shows covariate balance and propensity score distributions.

**Figure 2:**
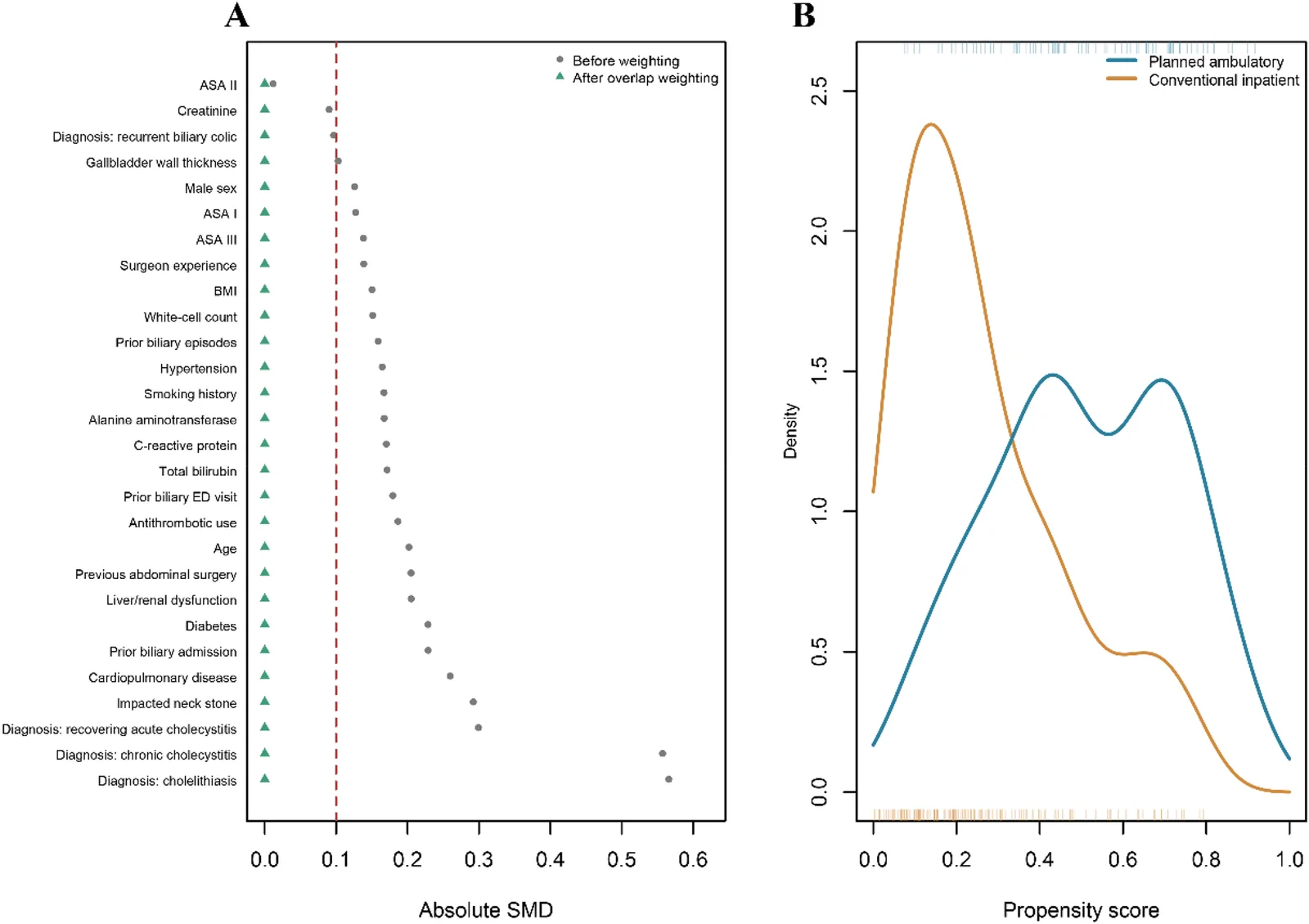
Covariate balance and common support for propensity-score overlap weighting. **(A)** Absolute standardised mean differences for baseline variables before and after weighting; the red dashed line marks the 0.10 balance threshold. **(B)** Kernel density distributions and observed propensity scores for the two pathways. All covariates had absolute standardised mean differences below 0.10 after overlap weighting.

### Operative course and early recovery

As shown in Table 2, morning surgery was significantly more common in the planned ambulatory group. The raw proportions were 87.2% and 52.4%, giving a weighted risk difference of 31.7 percentage points (95% confidence interval, 22.4–41.3; *P* < 0.001). Prophylactic antibiotic administration rates were similar, suggesting that this routine preventive measure was not a major source of confounding between pathway.

**Table 2:** Operative characteristics and early recovery.

Outcome	Ambulatory, unweighted	Inpatient, unweighted	Ambulatory, weighted	Inpatient, weighted	Effect (95% CI)	*P*-value
Morning surgery	68/78 (87.2%)	76/145 (52.4%)	84.5%	52.8%	RR 1.60 (1.37 to 1.90)	<0.001
Prophylactic antibiotics	74/78 (94.9%)	137/145 (94.5%)	95.7%	93.5%	RR 1.02 (0.95 to 1.11)	0.541
Anaesthesia duration, min	89.0 (81.0–98.0)	111.0 (100.0–120.0)	90.97	109.71	MD −18.74 (−23.37 to −14.71)	<0.001
Operating time, min	59.0 (49.0–68.0)	79.0 (69.0–88.0)	59.78	78.36	MD −18.58 (−22.05 to −15.48)	<0.001
Blood loss, mL	18.5 (13.0–24.2)	33.0 (26.0–45.0)	20.30	38.26	MD −17.96 (−23.14 to −14.15)	<0.001
Operative difficulty grade ≥2	18/78 (23.1%)	87/145 (60.0%)	27.1%	54.2%	RR 0.50 (0.31 to 0.67)	<0.001
Gallbladder perforation	4/78 (5.1%)	14/145 (9.7%)	7.8%	7.6%	RR 1.03 (0.35 to 2.35)	0.936
Bile or stone spillage	5/78 (6.4%)	18/145 (12.4%)	10.0%	10.2%	RR 0.98 (0.37 to 2.00)	0.957
Intraoperative bile duct injury	0/78 (0.0%)	1/145 (0.7%)	0.0%	0.7%	NE	0.449
Drain placement	8/78 (10.3%)	55/145 (37.9%)	14.9%	30.3%	RR 0.49 (0.25 to 0.73)	<0.001
Conversion to open surgery	0/78 (0.0%)	3/145 (2.1%)	0.0%	2.1%	NE	0.206
Post-anaesthesia care duration, min	60.5 (51.0–71.8)	74.0 (65.0–83.0)	62.09	74.13	MD −12.04 (−16.82 to −7.76)	<0.001
Discharge pain NRS	2.0 (2.0–3.0)	3.0 (3.0–4.0)	2.43	3.56	MD −1.13 (−1.47 to −0.83)	<0.001
Rescue analgesia	12/78 (15.4%)	41/145 (28.3%)	14.9%	26.0%	RR 0.57 (0.26 to 1.06)	0.083
Postoperative nausea and vomiting	8/78 (10.3%)	30/145 (20.7%)	11.4%	20.3%	RR 0.56 (0.21 to 1.12)	0.096
Time to ambulation, h	5.1 (4.2–5.9)	10.9 (8.8–12.7)	5.09	10.78	MD −5.69 (−6.44 to −5.02)	<0.001
Time to oral intake, h	5.5 (4.5–6.5)	12.9 (10.9–15.1)	5.84	12.78	MD −6.94 (−7.68 to −6.04)	<0.001
Time to discharge readiness, h	16.1 (14.1–18.7)	34.6 (30.1–41.5)	16.82	35.16	MD −18.34 (−20.44 to −16.23)	<0.001
Ambulatory pathway failure in source data	7/78 (9.0%)	Not applicable	Not applicable	Not applicable	Descriptive only	NE

Raw continuous outcomes are median (interquartile range); weighted columns show means or risks. Effects are mean differences (MDs) or risk ratios (RRs). Ambulatory failure resulted from pain (*n* = 2), postoperative nausea and vomiting (*n* = 2), drainage observation (*n* = 2), or delayed recovery (*n* = 1). Operative and recovery differences should not all be interpreted as direct causal effects. *P*-values correspond to the effect estimates shown in the “Effect (95% CI)” column: risk ratios for binary outcomes and mean differences for continuous outcomes.

After weighting, ambulatory care was associated with 18.7 fewer minutes of anaesthesia, 18.6 fewer operating minutes, and 18.0 mL less estimated blood loss (adjusted mean differences; all *P* < 0.001). Moderate-to-severe operative difficulty (Sugrue grade 2 or 3) and prophylactic drain placement were also less frequent. Because these events occurred or were confirmed after pathway assignment, they may reflect workflow differences, incompletely measured baseline selection, or both. The persistence of large differences in operative difficulty, operating time, estimated blood loss, and drain placement despite balance in the recorded preoperative covariates is consistent with residual clinical selection, particularly selection based on anticipated technical complexity that was not fully represented in the analytic dataset.

Early functional recovery generally favoured the ambulatory group. Weighted mean discharge pain scores were 2.43 and 3.56, representing an adjusted mean difference of −1.13 points (95% confidence interval, −1.47 to −0.83). Ambulation, oral intake and discharge readiness occurred 5.69, 6.94 and 18.34 h earlier; all *P* < 0.001. Rescue analgesia and postoperative nausea and vomiting had favourable point estimates, but their confidence intervals crossed the null. Seven ambulatory patients (9.0%) had pathway failure because of uncontrolled pain (*n* = 2), protracted nausea and vomiting (*n* = 2), prolonged peritoneal drainage observation (*n* = 2), or delayed functional recovery (*n* = 1).

### Thirty-day safety outcomes

Table 3 shows that the unweighted primary composite outcome occurred in four of 78 ambulatory patients (5.1%) and 16 of 145 inpatients (11.0%). Weighted risks were 3.8% and 10.2%, giving a risk ratio of 0.37 (95% confidence interval, 0.02 to 1.25) and a risk difference of −6.4 percentage points (−13.8 to 1.4; *P* = 0.100). The confidence interval for the risk difference included both lower and higher risk with ambulatory care, and the findings do not establish safety equivalence or non-inferiority.

**Table 3:** Perioperative and 30-day safety outcomes.

Outcome	Ambulatory, unweighted	Inpatient, unweighted	Ambulatory weighted risk	Inpatient weighted risk	Risk ratio (95% CI)	Risk difference (95% CI)	*P*-value (RD)
30-day adverse clinical composite	4/78 (5.1%)	16/145 (11.0%)	3.8%	10.2%	0.37 (0.02 to 1.25)	−6.4 (−13.8 to 1.4)	0.100
Any 30-day complication	3/78 (3.8%)	12/145 (8.3%)	3.0%	8.2%	0.37 (0.00 to 1.41)	−5.2 (−11.7 to 1.7)	0.135
Clavien–Dindo grade ≥2 complication	3/78 (3.8%)	12/145 (8.3%)	3.0%	8.2%	0.37 (0.00 to 1.41)	−5.2 (−11.7 to 1.7)	0.135
30-day emergency visit	2/78 (2.6%)	8/145 (5.5%)	1.7%	4.8%	0.36 (0.00 to 2.50)	−3.1 (−8.0 to 2.1)	0.240
30-day readmission	1/78 (1.3%)	5/145 (3.4%)	0.8%	4.3%	0.18 (0.00 to 1.59)	−3.6 (−8.5 to 0.9)	0.136
30-day ERCP or other intervention	1/78 (1.3%)	4/145 (2.8%)	1.3%	1.7%	0.76 (0.00 to 20.01)	−0.4 (−4.2 to 3.3)	0.824
Any 30-day re-intervention	1/78 (1.3%)	4/145 (2.8%)	1.3%	1.7%	0.76 (0.00 to 20.01)	−0.4 (−4.2 to 3.3)	0.824
30-day reoperation	0/78 (0.0%)	1/145 (0.7%)	0.0%	0.3%	NE	−0.3 (−2.1 to 0.0)	0.569
Intraoperative bile duct injury	0/78 (0.0%)	1/145 (0.7%)	0.0%	0.7%	NE	−0.7 (−3.1 to 0.0)	0.449
Conversion to open surgery	0/78 (0.0%)	3/145 (2.1%)	0.0%	2.1%	NE	−2.1 (−6.0 to 0.0)	0.206
30-day mortality	0/78 (0.0%)	0/145 (0.0%)	0.0%	0.0%	NE	0.0 (0.0 to 0.0)	NE

Weighted risks refer to the target propensity score overlap weighting population. Risk differences are expressed in percentage points. The primary composite endpoint included a Clavien–Dindo grade ≥2 complication, unplanned emergency department visit, unplanned hospital readmission, percutaneous or endoscopic intervention, unplanned reoperation, or all-cause death. NE, not estimable. Secondary *P* values are exploratory. *P*-values correspond to the risk difference (testing H₀: risk difference = 0) and were derived from non-parametric bootstrap standard errors. “Any 30-day complication” and “Clavien–Dindo grade ≥2 complication” show identical counts because no grade I complications were recorded in the dataset; minor (grade 1) events are likely under-ascertained in the retrospective database because they typically require no intervention and may not trigger a formal complication entry.

Any 30-day complication and a Clavien–Dindo grade ≥2 complication each occurred in three ambulatory patients and 12 inpatients. Overlap-weighted risks were 3.0% and 8.2%, producing a risk difference of −5.2 percentage points (95% confidence interval, −11.7 to 1.7; *P* = 0.135). Point estimates for emergency department visits, readmission, and unplanned reoperation or reintervention likewise did not suggest higher ambulatory risk, although all analyses were limited by low event rates and wide confidence intervals.

Supplementary Table S2 presents the available patient-level records for the 20 patients meeting the primary composite endpoint. Fifteen patients had a recorded Clavien–Dindo grade ≥2 complication, including three in the ambulatory group and 12 in the inpatient group. The remaining five patients, one in the ambulatory group and four in the inpatient group, met other component criteria despite having no recorded complication diagnosis or grade in the institutional dataset. Multiple component events could occur in the same patient, although each patient was counted only once in the composite analysis.

Only one bile duct injury, three conversions to open cholecystectomy, and one reoperation occurred, and no patient died within 30 days. Treatment effects for these rare outcomes were either inestimable or highly unstable. A non-significant statistical test should therefore not replace clinical judgement about these critical surgical events. Figure 3 summarises estimable safety outcomes using risk differences and 95% confidence intervals.

**Figure 3:**
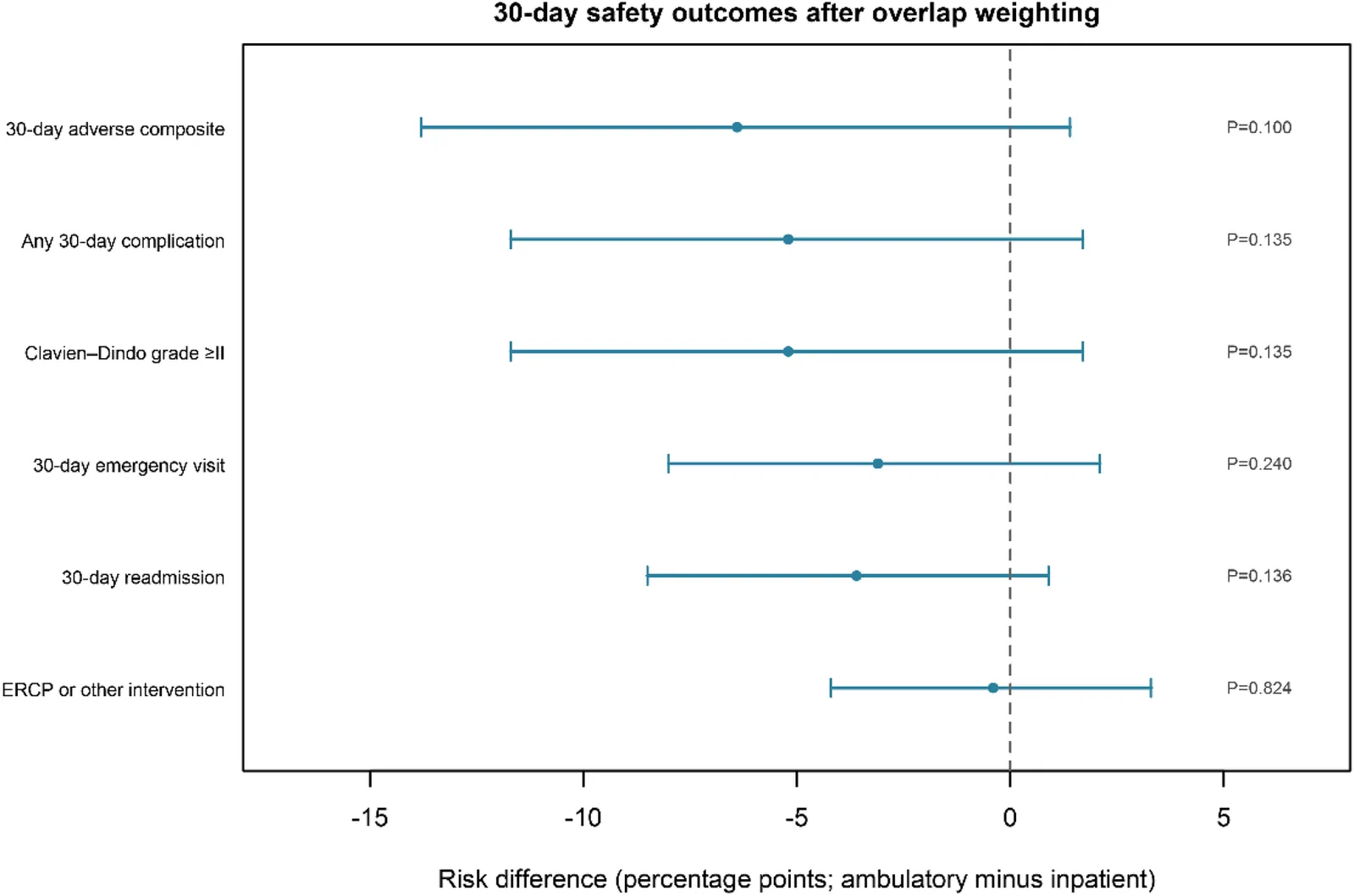
Thirty-day safety outcomes after overlap weighting. Points show weighted risk differences for planned ambulatory minus conventional inpatient care, and horizontal lines show 95% confidence intervals. Negative values favour ambulatory care. Confidence intervals used 5,000 patient-level non-parametric bootstrap resamples with refitting of the propensity score model in every sample.

### Index admission resource use and recorded hospital charges

As shown in Table 4, planned ambulatory care was associated with substantially lower index-admission resource utilisation. Weighted postoperative lengths of stays were 18.92 and 89.26 h, an adjusted difference of −70.33 h (95% confidence interval, −75.71 to −64.74). Weighted total hospital lengths of stays were 22.86 and 122.87 h, representing an adjusted mean difference of −100.01 h (−105.85 to −93.59). Both comparisons had *P* < 0.001. Because intended discharge within 24 h was a defining feature of the ambulatory pathway, these large differences in postoperative and total hospital lengths of stay were partly expected by design and are interpreted primarily as pathway-level resource utilisation outcomes rather than evidence of intrinsically faster physiological recovery.

**Table 4:** Length of stay and recorded healthcare resource use.

Outcome	Ambulatory, unweighted	Inpatient, unweighted	Ambulatory, weighted	Inpatient, weighted	Effect (95% CI)	*P*-value
Postoperative stay, h	17.1 (16.3–19.0)	87.4 (70.7–107.7)	18.92	89.26	MD −70.33 (−75.71 to −64.74)	<0.001
Total hospital stay, h	21.4 (20.4–22.6)	122.3 (103.4–140.6)	22.86	122.87	MD −100.01 (−105.85 to −93.59)	<0.001
Medication charges, CNY	845.5 (747.8–960.8)	3,296.0 (2,920.0–3,822.0)	913.85	3,409.98	MD −2,496.14 (−2,650.11 to −2,344.18)	<0.001
Surgical and anaesthetic charges, CNY	4,826.5 (4,594.8–5,076.5)	7,414.0 (7,181.0–7,744.0)	4,845.39	7,549.70	MD −2,704.31 (−2,895.81 to −2,549.21)	<0.001
Recorded aggregate index admission hospital charges, CNY	5,687.0 (5,506.8–6,000.0)	10,799.0 (10,305.0–11,345.0)	5,759.24	10,959.68	MD −5,200.44 (−5,484.33 to −4,965.46)	<0.001
Unplanned healthcare encounters within 30 days	0.0 (0.0–0.0)	0.0 (0.0–0.0)	0.04	0.11	MD −0.07 (−0.18 to 0.02)	0.173

Raw unadjusted values are median (interquartile range), weighted values are means, and effects are adjusted mean differences. Recorded aggregate index-admission hospital charges equal the available medication charges and surgical and anaesthetic charges. These amounts were obtained from the institutional billing system and do not represent comprehensive hospital cost-accounting expenditures, third-party payer reimbursement amounts, or a formal economic evaluation. *P* values correspond to the mean differences shown in the “Effect (95% CI)” column.

Medication charges were CNY 2,496 lower, surgical and anaesthetic charges were CNY 2,704 lower, and aggregate recorded index-admission hospital charges were CNY 5,200 lower (adjusted mean differences; all *P* < 0.001. Unplanned healthcare encounters within 30 days did not differ significantly between groups. Figure 4 shows adjusted differences in hospital-use time and recorded hospital length of stay. The between-group difference in recorded hospital charges partly reflected the institutional tariff structure: the ambulatory pathway was billed under the day surgery operating theatre and anaesthetic tariff, whereas the conventional pathway was billed under the inpatient tariff, which included additional charges related to extended post-anaesthesia care and postoperative monitoring.

**Figure 4:**
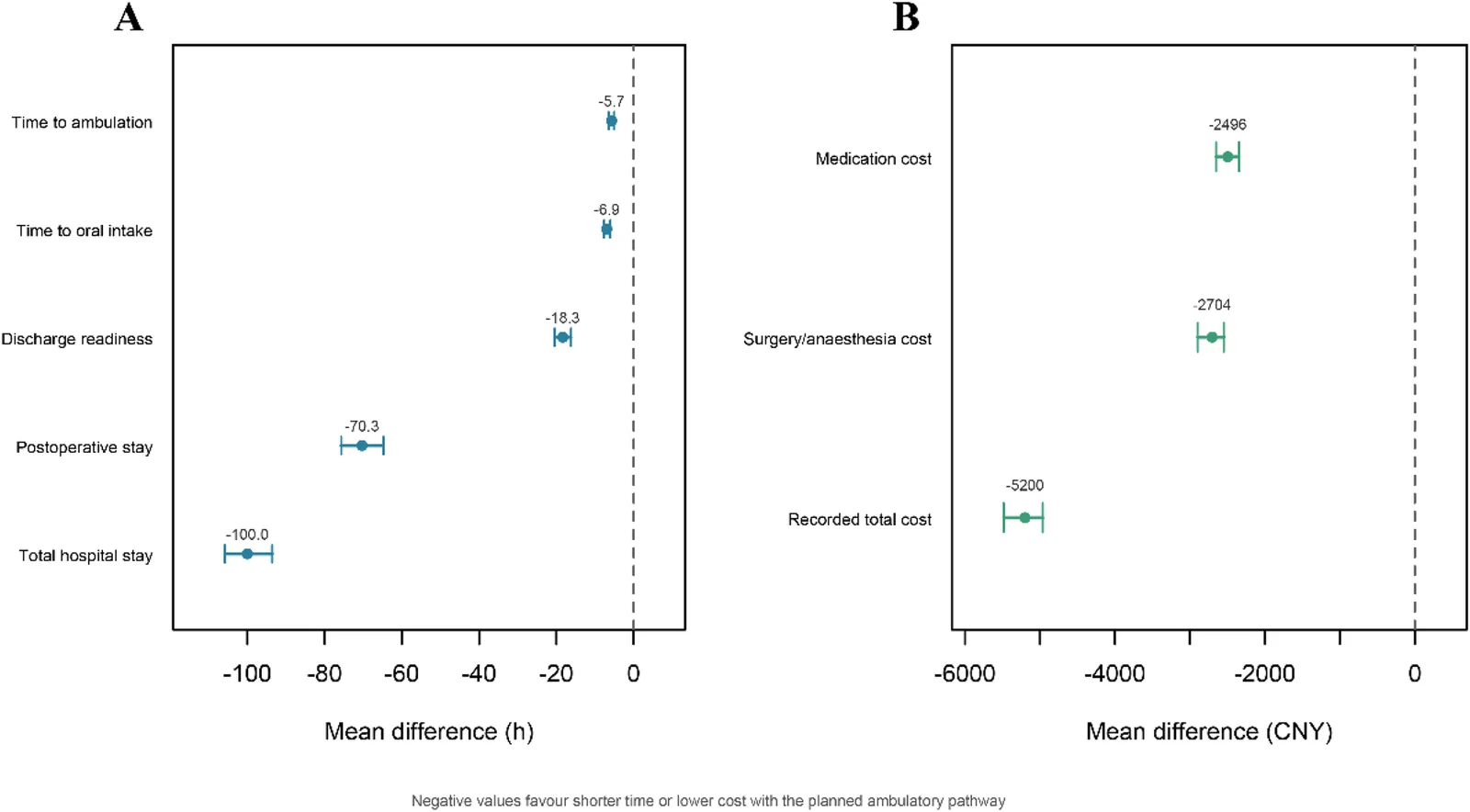
Recovery time, hospital length of stay, and recorded charge differences after overlap weighting. **(A)** Adjusted mean differences in time to ambulation, oral intake and discharge readiness, postoperative length of stay and total hospital length of stay. **(B)** Adjusted mean differences in medication charges, surgical and anaesthetic charges, and aggregate recorded index-admission hospital charges. Points show mean differences and horizontal lines show 95% confidence intervals; negative values indicate shorter durations or lower recorded charges with ambulatory care.

### Sensitivity analysis

Fifty-nine ambulatory patients were successfully matched 1:1 to 59 inpatients within the pre-specified calliper. Primary composite event rates were 5.1% and 8.5%, giving a risk difference of −3.4 percentage points (95% confidence interval, −11.9 to 5.1; *P* = 0.475). This result was directionally consistent with the overlap-weighted analysis, although statistical uncertainty remained substantial given the reduced effective sample size.

In the matched cohort, adjusted mean differences were −68.2 h for postoperative length of stay, −97.8 h for total hospital length of stay, and CNY −5,144 for recorded aggregate index-admission hospital charges; all *P* < 0.001. The persistence of comparable resource utilisation estimates across two propensity score approaches reduces the likelihood that these findings depended entirely on a single weighting specification.

## Discussion

### Principal findings

In this single-centre cohort, propensity score overlap weighting achieved close balance in measured preoperative characteristics. However, pathway assignment also depended on clinical and organisational factors that were not systematically recorded, balance in measured covariates should therefore not be interpreted as elimination of pathway selection bias. Planned ambulatory laparoscopic cholecystectomy was not associated with an observed increase in the 30-day adverse clinical composite endpoint. It was also associated with earlier recorded functional recovery milestones, shorter hospital lengths of stay, and lower recorded index-admission hospital charges. Sensitivity analysis using 1:1 propensity score matching supported the direction and magnitude of these findings. Nevertheless, these estimate represent an observational association within a clinically selected overlap population rather than a definitive causal effect of the pathway.

The statistical evidence was stronger and more precise for resource utilisation differences than for the safety composite endpoint. Nevertheless, both safety and resource estimates remain susceptible to residual confounding because important clinical and non-clinical determinants of pathway eligibility were not fully measured. The study therefore describes associations between planned clinical pathways and observed outcomes and does not establish safety equivalence or causal effects of ambulatory care.

### Safety and patient selection

Our results align with the general direction of previous randomised trials, cohort studies and meta-analyses. Ambulatory laparoscopic cholecystectomy is not shown a clear increase in serious complications or unplanned readmission among carefully selected patients (9–19). Recent reviews further indicate that pathway success does not depend on an isolated age or ASA physical status threshold. Baseline comorbid risk, operating time, intraoperative surgical findings, early recovery trajectory, and organisational capacity act together (20–23).

The 9.0% pathway failure rate illustrates that a structured ambulatory programme requires predefined escalation criteria and rescue overnight bed capacity rather than assuming that every patient will achieve planned same-day discharge. Retaining delayed discharges as ambulatory failures avoids moving poor pathway performance into the inpatient comparator. Clinically, explicit escalation criteria and guaranteed access to unplanned overnight care matter more than an unrealistically perfect same-day discharge rate.

Rare serious adverse events still deserve separate clinical attention. Death, iatrogenic bile duct injury, conversion to open surgery, unplanned reoperation, and percutaneous or endoscopic intervention were infrequent in the observed cohort, but the small event counts preclude precise between-group inference for these individual outcomes. Prevention of bile duct injury continues to depend on safe anatomical dissection, alternative operative strategies when needed, and timely intraoperative escalation (3–6). Reliable post-discharge monitoring contact and rapid return pathways are also essential because readmission after laparoscopic cholecystectomy is uncommon but not negligible (33).

### Early recovery and perioperative organisation

Early functional recovery differences were larger and more consistent than safety differences. Previous work identifies inadequately controlled pain, postoperative nausea and vomiting, delayed mobilisation, and late theatre start times as the principal barriers to successful ambulatory discharge (12, 13, 20, 33–36). Ambulatory patients in this cohort were significantly more often operated on in the morning. They reached discharge readiness about 18 h earlier and mobilised and ate about six and seven hours earlier. Ambulatory surgery therefore appears to be an organised perioperative package rather than simply a shorter bed allocation.

The comparison also has an important causal boundary. Pathway assignment incorporated clinical and organisational judgements that were not fully represented in the analytic dataset. Functional capacity, anticipated technical complexity, home social support, patient understanding of the ambulatory pathway, telephone availability, and rapid emergency-return feasibility contributed to clinical selection but were not systematically recorded using structured instruments. Consequently, these factors could not be incorporated into the propensity score model.

Operating and anaesthesia duration, estimated blood loss, operative difficulty, prophylactic drain placement, postoperative pain and postoperative nausea, and vomiting occurred or became evident after pathway assignment and were therefore not appropriate baseline variables for propensity score adjustment. Nevertheless, the substantially lower operative difficulty, shorter operating time, lower estimated blood loss, and less frequent peritoneal drainage observed in the ambulatory group after weighting provide indirect evidence that clinicians selected technically less complex patients in ways that were incompletely captured by the recorded preoperative covariates.

Accordingly, overlap weighting should be interpreted as improving comparability with respect to measured baseline characteristics rather than eliminating selection bias. The resulting estimates are exploratory observational associations within the empirically defined overlap population and should not be interpreted as causal effects of ambulatory pathway assignment.

Multimodal opioid-sparing analgesia and combination effective prophylaxis for postoperative nausea and vomiting may facilitate early ambulatory recovery (36, 37). However, our database lacked granularity regarding specific drug doses, the use of regional nerve blocks, and protocol adherence measures, so no single perioperative intervention can explain the observed symptom differences. Future prospective studies should systematically record validated preoperative surgical difficulty scores, individual surgeons, reasons for pathway assignment, postoperative symptoms at fixed time points, and validated instruments measuring patient-reported recovery quality.

### Healthcare resource implications

The observed resource differences have direct organisational relevance. However, the large reduction in hospital length of stay was partly expected by design, as discharge within 24 h was a defining operational feature of the ambulatory pathway. Accordingly, these stay estimates should primarily be interpreted as measures of pathway-level hospital resource utilisation rather than as evidence of intrinsically faster physiological recovery or greater medical efficiency. Overlap-weighted estimates corresponded to about 70 fewer postoperative hours and 100 fewer total hospital hours per ambulatory patient, potentially releasing several bed-days. At high-volume centres, such differences may affect elective throughput and bed availability. Previous randomised trials, meta-analyses and systematic reviews similarly link structured ambulatory pathways with shorter hospital stays after laparoscopic cholecystectomy (9–11, 15, 18, 20, 34, 38–43).

The monetary findings require a narrower interpretation. The reported amounts were recorded as index-admission hospital charges obtained from the institutional billing system rather than comprehensive hospital cost-accounting estimates or third-party payer reimbursement amounts. The available aggregate measure comprised medication charges and surgical and anaesthetic charges but did not comprehensively capture ward and nursing services, diagnostic investigations, administrative overhead, post-discharge ambulatory care, patient travel, informal caregiving, or other patient-borne costs. In addition, the between-group difference partly reflected the institutional day surgery and inpatient tariff structures. These findings therefore indicate lower recorded index-admission hospital charges within this specific institution and should not be interpreted as evidence of formal cost-effectiveness, complete healthcare-system savings, or societal economic benefit.

### Strengths and limitations

Several design features strengthen the study. First, group assignment followed the preoperative plan, and delayed discharge remained an ambulatory failure, reducing bias from classification by realised length of stay. Second, the patient-level primary composite endpoint integrated clinically important adverse events and was reported with absolute and relative effect estimates and confidence intervals. Third, bounded overlap weights achieved strong measured balance, while 1:1 calliper matching provided an independent sensitivity analysis.

This study has several important limitations. As a single-centre retrospective cohort study, it remains susceptible to measurement error, selection bias, and unmeasured confounding. Propensity score methods balance only recorded covariates (24, 27), whereas functional status, anticipated operative complexity, and social or logistical determinants of pathway eligibility were incompletely captured; persistent differences in operative characteristics after weighting suggest residual clinical selection by indication. Missing patient-level operation dates and surgeon identifiers precluded adjustment for calendar time trends and surgeon-specific practice or clustering, and surgeon experience category alone could not fully capture these effects. In addition, several recovery outcomes were partly determined by the pathway protocol itself: time to mobilisation, oral intake, and hospital length of stay may reflect scheduled care processes rather than faster biological recovery. Although postoperative assessment criteria and intervals were identical for both groups, discharge pain was measured at different postoperative time points (day of surgery vs. postoperative day 1 or later) and is not directly comparable. The conventional inpatient pathway followed a traditional overnight observation policy with prolonged preoperative workup and administrative waiting time; the observed length of stay advantage may therefore be substantially smaller in centres employing modern enhanced recovery after surgery or short-stay overnight protocols. The reported direct medical cost was limited to aggregate surgical, anaesthetic, and medication charges, did not include ward, nursing, diagnostic, administrative, post-discharge, or patient-borne costs, and reflected institutional tariff differences rather than resource consumption alone; the true economic difference may differ substantially from the estimate reported here. Outcome ascertainment and severity assessment were limited by incomplete records: follow-up sources, telephone protocols, external facility care ascertainment, and assessor blinding could not be fully verified, and differential under-ascertainment between pathways cannot be excluded. Missing event dates and treatment details prevented independent verification of Clavien–Dindo grades; no grade I complications were recorded, and the absence of such minor events likely reflects incomplete ascertainment in the retrospective database rather than a true zero rate. Finally, the composite outcome combines events of differing clinical importance and should not be interpreted as a uniform measure of serious morbidity, and without a pre-specified non-inferiority margin, a non-significant comparison cannot establish safety equivalence. The findings therefore represent exploratory associations rather than causal treatment effects.

## Conclusions

Among selected adults undergoing elective laparoscopic cholecystectomy surgery for benign gallstone-related disease who had a realistic possibility of receiving either pathway, planned ambulatory care was associated with no observed increase in the 30-day adverse clinical composite endpoint, and with shorter hospital lengths of stay and lower recorded index-admission hospital charges. However, critical clinical and logistical important determinants of pathway eligibility were not fully captured in the institutional database, and substantial residual confounding by indication may remain. These findings should therefore be interpreted as exploratory observational associations in a clinically selected overlap population and do not establish causal treatment effects, safety equivalence, formal non-inferiority or justification for indiscriminate expansion of ambulatory eligibility.

## Data Availability

The raw data supporting the conclusions of this article will be made available by the authors, without undue reservation.
