# Supplementary material for: Thirty-day clinical outcomes, early recovery, and healthcare resource use after planned ambulatory vs. conventional inpatient laparoscopic cholecystectomy: a propensity-score overlap-weighted retrospective cohort study
Source: Front Surg. 2026 Oct 16;13:1962424. doi: 10.3389/fsurg.2026.1962424 (PMC13719444; doi:10.3389/fsurg.2026.1962424)
Supplement: Supplementary file 1 [file Table1.docx]

**Table S1**. Distribution of planned surgical pathways across available calendar periods

| **Calendar period** | **Total procedures, n** | **Planned ambulatory, n (%)** | **Conventional inpatient, n (%)** |
| --- | --- | --- | --- |
| May–July 2025 | 49 | 13 (26.5%) | 36 (73.5%) |
| August–October 2025 | 57 | 26 (45.6%) | 31 (54.4%) |
| November 2025–January 2026 | 31 | 12 (38.7%) | 19 (61.3%) |
| February–June 2026 | 86 | 27 (31.4%) | 59 (68.6%) |
| **Total** | **223** | **78 (35.0%)** | **145 (65.0%)** |

Values are counts and percentages within each calendar period. The institutional ambulatory laparoscopic cholecystectomy programme began in January 2025. Exact patient-level operation dates were not retained in the analytic dataset; therefore, these aggregate calendar-period counts were used descriptively.

**Table S2**. Patient-level records for the 20 patients with the 30-day adverse clinical composite

| **ID** | **Planned pathway** | **Recorded complication diagnosis** | **Recorded CD grade** | **Other recorded composite components** |
| --- | --- | --- | --- | --- |
| 1 | Ambulatory | Pulmonary infection | II | ED visit |
| 2 | Ambulatory | Intra-abdominal infection | IIIa | Readmission |
| 3 | Ambulatory | NR | NR | ED visit |
| 4 | Ambulatory | Surgical-site infection | II | Intervention |
| 5 | Inpatient | Urinary retention | II | Readmission |
| 6 | Inpatient | Surgical-site infection | II | None recorded |
| 7 | Inpatient | Pulmonary infection | II | Readmission |
| 8 | Inpatient | Surgical-site infection | II | None recorded |
| 9 | Inpatient | Surgical-site infection | II | None recorded |
| 10 | Inpatient | NR | NR | ED visit; Readmission |
| 11 | Inpatient | NR | NR | ED visit; Readmission; Intervention |
| 12 | Inpatient | Intra-abdominal infection | IIIa | ED visit |
| 13 | Inpatient | Urinary retention | IIIa | ED visit |
| 14 | Inpatient | Intra-abdominal infection | IIIa | ED visit; Intervention |
| 15 | Inpatient | NR | NR | ED visit; Intervention |
| 16 | Inpatient | Intra-abdominal bleeding | IIIb | ED visit; Readmission |
| 17 | Inpatient | Pulmonary infection | II | None recorded |
| 18 | Inpatient | NR | NR | ED visit; Intervention; Reoperation |
| 19 | Inpatient | Surgical-site infection | II | None recorded |
| 20 | Inpatient | Pulmonary infection | II | None recorded |

CD, Clavien–Dindo; ED, emergency department; NR, not recorded. Inpatient denotes the conventional inpatient pathway. Intervention denotes the source category “ERCP or other intervention”.

**Table S3**. Propensity-score model specification, stability, and balance diagnostics for overlap weighting and 1:1 matching

**Panel A**. Propensity-score model specification

| Covariate | Variable type | Coding / functional form | Nonlinear terms or interactions |
| --- | --- | --- | --- |
| Age | Continuous | Linear (untransformed), years | None |
| Sex | Binary | Male = 1, Female = 0 | None |
| BMI | Continuous | Linear (untransformed), kg/m² | None |
| Smoking history | Binary | Yes = 1, No = 0 | None |
| ASA class | Multicategory (3 levels) | Indicator coding; ASA I = reference; ASA II and ASA III as separate 0/1 indicators | None |
| Hypertension | Binary | Yes = 1, No = 0 | None |
| Diabetes | Binary | Yes = 1, No = 0 | None |
| Cardiopulmonary disease | Binary | Present = 1, Absent = 0 | None |
| Liver or renal dysfunction | Binary | Present = 1, Absent = 0 | None |
| Antithrombotic therapy | Binary | Yes = 1, No = 0 | None |
| Previous abdominal surgery | Binary | Yes = 1, No = 0 | None |
| Primary diagnosis | Multicategory (4 levels) | Indicator coding; cholelithiasis = reference; chronic calculous cholecystitis, recurrent biliary colic, and recovering mild acute cholecystitis as separate 0/1 indicators | None |
| Previous biliary episodes | Continuous | Linear (untransformed), number of episodes | None |
| Previous biliary emergency visit | Binary | Yes = 1, No = 0 | None |
| Previous biliary admission | Binary | Yes = 1, No = 0 | None |
| Gallbladder wall thickness | Continuous | Linear (untransformed), mm | None |
| Impacted gallbladder neck stone | Binary | Yes = 1, No = 0 | None |
| White blood cell count | Continuous | Linear (untransformed), ×10⁹/L | None |
| C-reactive protein | Continuous | Linear (untransformed), mg/L | None |
| Alanine aminotransferase | Continuous | Linear (untransformed), U/L | None |
| Total bilirubin | Continuous | Linear (untransformed), μmol/L | None |
| Creatinine | Continuous | Linear (untransformed), μmol/L | None |
| Surgeon experience | Continuous | Linear (untransformed), years | None |

**Panel B**. Model stability and goodness-of-fit

| Metric | Value |
| --- | --- |
| Total sample size, n | 223 |
| Ambulatory (treated), n | 78 |
| Conventional inpatient (control), n | 145 |
| Number of model parameters (excluding intercept) | 26 |
| Events-per-variable (EPV), treated ÷ parameters | 78 / 26 = 3.00 |
| Model convergence | Converged; no warning or error |
| Quasi-complete or complete separation in original fit | None detected |
| Firth penalised regression used | No (standard maximum-likelihood logistic regression) |
| Firth penalised refit as sensitivity check | Yes; PS estimates highly correlated with standard fit (Pearson r = 0.986); conclusions unchanged |
| Hosmer–Lemeshow goodness-of-fit, χ² (df = 8) | 7.14 (p = 0.521) |
| C-statistic (AUC) | 0.79 (95% CI: 0.73–0.85) |
| Variance inflation factors (VIFs), range | 1.04–2.87 |
| Maximum VIF (variable) | 2.87 (Previous biliary episodes) |
| All VIFs < 5 | Yes |
| Propensity score range, ambulatory | 0.076–0.917 |
| Propensity score range, inpatient | 0.002–0.793 |
| Overlap weight range | 0.002–0.924 |
| Proportion of weights < 0.01 | 0.0% (0 / 223) |
| Effective sample size, ambulatory | 65.1 |
| Effective sample size, inpatient | 92.7 |
| Effective sample size ratio (ESS / original n), ambulatory | 65.1 / 78 = 83.5% |
| Effective sample size ratio (ESS / original n), inpatient | 92.7 / 145 = 63.9% |

**Panel C**. Covariate balance diagnostics before and after overlap weighting and 1:1 matching

| **Covariate** | **Pre-weight absolute SMD** | **Post-OW SMD** |
| --- | --- | --- |
| Age | **0.202** | 0.000 |
| BMI | **0.150** | 0.000 |
| Previous biliary episodes | **0.159** | 0.000 |
| Gallbladder wall thickness | **0.103** | 0.000 |
| White blood cell count | **0.151** | 0.000 |
| C-reactive protein | **0.170** | 0.000 |
| Alanine aminotransferase | **0.167** | 0.000 |
| Total bilirubin | **0.171** | 0.000 |
| Creatinine | **0.090** | 0.000 |
| Surgeon experience | **0.139** | 0.000 |
| Male sex | **0.126** | 0.000 |
| Smoking history | **0.167** | 0.000 |
| Previous abdominal surgery | **0.205** | 0.000 |
| Hypertension | **0.165** | 0.000 |
| Diabetes | **0.229** | 0.000 |
| Cardiopulmonary disease | **0.260** | 0.000 |
| Liver/renal dysfunction | **0.205** | 0.000 |
| Antithrombotic therapy | **0.186** | 0.000 |
| Previous biliary emergency visit | **0.179** | 0.000 |
| Previous biliary admission | **0.229** | 0.000 |
| Impacted gallbladder neck stone | **0.292** | 0.000 |
| Cholelithiasis | **0.566** | 0.000 |
| Chronic calculous cholecystitis | **0.557** | 0.000 |
| Recurrent biliary colic | **0.096** | 0.000 |
| Recovering mild acute cholecystitis | **0.299** | 0.000 |

**Panel D**. Bootstrap performance diagnostics (overlap weighting)

| Diagnostic | Result |
| --- | --- |
| Number of bootstrap replications | 5,000 |
| Resampling unit | Patient (n = 223 resampled with replacement per replicate) |
| Propensity model re-fitted within each bootstrap sample | Yes |
| Outcome analysis re-run within each bootstrap sample | Yes |
| Bootstrap random seed | 20250501 |
| Replicates with perfect or quasi-complete separation | 3 (0.06%) |
| Replicates with non-convergence (optimiser warning) | 7 (0.14%) |
| Replicates with zero ambulatory events (primary composite) | 42 (0.84%) |
| Replicates with zero inpatient events (primary composite) | 0 (0.00%) |
| Replicates with zero events in either group | 42 (0.84%) |
| Total flagged replicates (separation + non-convergence + zero-event) | 52 (1.04%) |
| Handling of flagged replicates | Excluded from percentile calculation; sensitivity analysis including them with Firth penalised refit yielded near-identical point estimates and CIs |
| Median (IQR) number of matched pairs across bootstrap replicates (matching sensitivity) | 59 (57–61) |
| Confidence interval type | Percentile (empirical 2.5th and 97.5th percentiles of the bootstrap distribution) |
| Bias-corrected (BCa) intervals computed as sensitivity | Yes; BCa intervals were within ±0.3 percentage points of percentile intervals for all primary outcomes |
| Bootstrap standard error used for two-sided P values | Yes |

### **Panel E**. 1:1 Matching sensitivity analysis — specifications and diagnostics

| **Specification** | **Detail** |
| --- | --- |
| Matching method | 1:1 nearest-neighbour matching without replacement |
| Distance metric | Propensity-score logit |
| Caliper | 0.20 × SD of the propensity-score logit |
| Caliper width (on logit scale) | 0.20 × 1.48 = 0.296 |
| Matched pairs, n | 59 |
| Ambulatory patients matched, n (%) | 59 / 78 (75.6%) |
| Unmatched ambulatory patients, n | 19 |
| Unmatched inpatient patients, n | 86 |
| Estimand | Sample average treatment effect on the treated (SATT) |
| Target population | The subset of ambulatory patients who could be matched to a comparable inpatient (not the full ambulatory group) |
| Post-matching balance: covariates with SMD < 0.10 | 22 / 24 (91.7%) |
| Post-matching balance: covariates with SMD ≥ 0.10 | 2 (surgeon experience, 0.114; previous biliary episodes, 0.127) |
| Post-matching variance ratios (continuous), range | 0.82–1.12 |
| Post-matching variance ratios, all within 0.5–2.0 | Yes |
| Variance estimation method | Paired (clustered) nonparametric bootstrap, 5,000 replicates, treating each matched pair as a cluster |
| Matching re-performed within each bootstrap replicate | Yes |
| Median (IQR) number of matched pairs across bootstrap replicates | 59 (57–61) |
| Confidence intervals (matching) | Percentile (empirical 2.5th and 97.5th percentiles) |
| Directional agreement with overlap-weighted estimates | Primary composite, postoperative stay, total stay, and total cost all showed the same direction of effect as overlap weighting |
| Interpretation caveat | Matching targets the SATT in the matched ambulatory subset; |
